# Self-Related Health, Physical Activity and Complaints in Swedish High School Students

**DOI:** 10.1100/tsw.2006.164

**Published:** 2006-07-18

**Authors:** Marie Alricsson, Bodil J. Landstad, Ulla Romild, Suzanne Werner

**Affiliations:** Department of Health Sciences, Mid Sweden University, Ostersund, Sweden; Faculty of Education, Engineering and Nursing, Nord-Trøndelag University College, Levanger, Norway; Department of Information Technology and Media, Mid Sweden University, Östersund, Sweden; Capio Artro Clinic, Stockholm Sports Trauma Research Center, Karolinska Institutet, Stockholm, Sweden; Department Neurotec, Division of Physiotherapy, Karolinska Institutet, Stockholm, Sweden

**Keywords:** adolescents, exertion, gender, questionnaire, sports, survey, symptoms, Sweden

## Abstract

The aim of this investigation was to study self-related health, physical activity and level of exertion, as well as body complaints in Swedish high school students. A total of 993 high school students aged 16–19 years participated in the study. A questionnaire was completed at school and included questions about self-related health, physical activity behavior, type of physical activity/sport, intensity, duration, possible injuries or complaints, and absence from physical training at school, during the last 3 months. The results showed that 26% of the high school students participated in sports on a regular basis. Males reported significantly better health than females (*p* < 0.0005). A significantly higher number of females participated in physical activities at a lower level of effort (*p* < 0.0005) and a higher number of males trained at a higher level of effort (*p* < 0.005). Sixtyone percent reported body pain during the last 3 months, representing a higher number of females than males (*p* = 0.03). A higher number of females than males reported complaints from the back (*p* = 0.002), the knees (*p* = 0.015), the neck (*p* = 0.001), and the hip (*p* = 0.015). Females with body complaints reported poorer health than those without complaints. There was a correlation between poor self-related health and a lower level of physical effort (0.219; *p* < 0.001). The results showed that the prevalence of musculoskeletal symptoms was high in this population and demonstrated a certain association with self-related health. Therefore, it is important to make it easy for adolescents to perform physical activity at school and during their leisure time in order to prevent chronic diseases.

## INTRODUCTION

Recent reports have found a strong correlation between the amount of physical activity and health in grown-up individuals. Physical inactivity is closely related to an increased risk of cardiovascular disease, diabetes mellitus, osteoporosis, and certain types of cancer[1,2,3,4,5]. Furthermore, it is reported that physical inactivity during growth might lead to impaired health later in life[6]. In Sweden, adolescents today are considerably less physically active compared with adolescents a decade ago. The majority of adolescents prefer to watch television and/or use the computer instead of participating in sports or physical activities. This is an important dilemma from a health point of view[7]. The findings about attitudes to sports and sociodemographic factors that impact on physical activity habits and fitness in adults are complex[6]. Wendell et al.[8] found that experiences related to participation in activity during childhood and adolescence may positively influence physical activity during adulthood. Furthermore, Telama et al.[9] reported that persistent participation in sports increases the probability of a higher level of physical activity later in life.

Back problems have been reported to occur as early as the age of 16 for both males and females. In a study comparing adolescents from 1974 with those from 1995, Westerstahl et al.[10] reported a decrease in aerobic fitness and an increase in maximal static strength. This development was partly due to an increase in body weight and body mass index (BMI). However, a decrease in daily physical activity may also present as a contributing factor in terms of decline in aerobic fitness. In their study from 1974–1995, a reduction of muscular endurance was not found to be related to the increase in body weight and BMI. On the contrary, the authors found that such decline was related to a reduction of hip flexion and arm muscle endurance[10].

During the last decade, there has been a paradigm shift towards recommendations of physical activity, with an emphasis being placed on physical activity in daily life[11]. The understanding of how the activity level in children and body composition are related regarding development and maintenance of a healthy lifestyle is thereby a growing area of research[12]. The International Consensus Conference in the U.S. on Physical Activity Guidelines for Adolescents convened to review the effects of physical activity on the health of adolescents. This meeting of delegates established age-appropriate physical activity guidelines, and considered how such guidelines could be implemented in primary health care settings. Thirty-four invited experts and representatives of scientific, medical, and governmental organizations established two main guidelines[13]. First, all adolescents should be physically active daily or nearly every day as part of their lifestyle. Second, adolescents should be engaged in three or more sessions per week of activities that last 20 min or more and that require medium to vigorous levels of exertion[13].

Further supporting these recommendations, the Norwegian physical activity recommendations for children and youth stated that there should be a minimum of 60 min for physical activity of at least a medium intensity every day. In addition, Klasson-Heggebo and Anderssen[14] evaluated, objectively, the level of physical activity in a representative cohort of urban children and youth in Norway. Their study was guided by these recommendations. The results indicated that 86% of the 9 year olds and 55% of the 15 year olds met these recommendations. Furthermore, the activity level was higher in males than in females[14].

The aim of the present investigation was to study self-related health, physical activity, and level of exertion, as well as body complaints in high school students in Sweden. Based on these aspects, another aim was to compare males and females, students in academic programs with those in vocational programs, and students living in different demographic locations (i.e., city vs. countryside).

## METHODS

A total of 993 students, 49% males and 51% females, from three different communities in the northern part of Sweden participated voluntarily in the study. Mean age of the students was 18.0 (± 1.0) years. In Sweden, high school students, grade 1, 2, and 3, are 16–19 years old. In the current city, there were two high schools. An inquiry to participate was sent to both schools and one of them accepted. An inquiry was also sent to the two nearest high schools in the countryside and both these schools accepted to participate.

A questionnaire was completed at school during school hours. The class mentor distributed and collected the questionnaires. The questionnaires were filled out and returned by all students present at this particular occasion, supervised by their class mentor, and then forwarded to the research leader for statistical evaluation.

## Questionnaire

The questionnaire included questions about self-related health, physical activity behavior, type of physical activity/sport, intensity, duration, possible injuries or complaints, and absence from physical training at school, during the last 3 months[15,16].

- **Self-related health —** Self-related health was determined based on the following three different questions: (1) "How do you judge your general health?", (2) "How do you rate your health?", and (3) "How do you find your general health when compared with other individuals of the same age?"
- **Physical activity behavior —** To investigate physical activity, the students reported how often they performed physical activity at a high, medium, or low level of effort. A high level was defined as increased pulse rate, breathlessness, and sweating. A medium level was defined as being able to talk with somebody during the physical performance, and a low level was defined as walking and/or cycling at a slow tempo.
- **Type of physical activity/sport —** The students were asked to report the types of activities they performed, and the frequency and duration of these activities during the last 6 months.
- **Symptoms or complaints —** Possible symptoms or complaints during the last 3 months were determined based on the following questions: "Have you during *the last 3 months* had any symptoms from any part of the body?" If "yes", they were further asked to specify which body part that was symptomatic.
- **Test-retest of the questionnaire —** The questionnaire has been tested showing a very good reliability[16].

## Statistical Analysis

The results of the questionnaire were analyzed using SPSS 12.0.1. Data were summarized in frequency tables and tested with Pearson's Chi-2-test of independence. Difference between groups was also tested with Students *t*-test for two-sample assuming equal variances. The level of significance was set at 5%.

## Ethical Considerations

The Swedish National Ethics Committee approved the study. The students participated voluntarily and the answers remained anonymous. Completion of the questionnaire was taken as consent. All questionnaires are de-identified, as they contain no information that enables individuals to be recognized. Statistical analysis will be undertaken and, therefore, the cohort will be reported on collective groups.

## RESULTS

Main characteristics of the participants are shown in Table 1. As shown in the table, 26% of the high school students participated in sports on a regular basis. The most popular sports among males were (in order of popularity) soccer, cycling, running, alpine skiing, and floor ball. Among females, the most popular sports were gymnastics, running, cycling, alpine skiing, and swimming. A great deal of those who did not perform in any sport spent time with friends, watched TV, used the computer, read books or magazines, listened to music, or played video games.

**TABLE 1**
**Main Characteristics of the Subjects (n = 993)**

| Characteristics | Male; n (%) | Female; n (%) | Total; n (%) |
|---|---|---|---|
| Academic program | 270 (43.0) | 361 (57.0) | 631 |
| Vocational program | 214 (59.1) | 148 (40.9) | 362 |
| City schools | 347 (46.5) | 399 (53.5) | 746 |
| Countryside schools | 137 (55.5) | 110 (44.5) | 247 |
| Body pain last 3 months | 277 (57) | 331 (65) | 608 (61) |
| Active in sports | 134 (28) | 126 (25) | 260 (26) |

### Self-Related Health

Males reported significantly better health than females ($p < 0.0005$); 81% of males and 71% of females reported their health to be good.

### Level of Physical Effort

A significantly higher number of females participated in physical activities at a lower level of effort one to three times per week or more ($p < 0.0005$). On the other hand, a significantly higher number of males trained at a higher level of effort one to three times per week or more ($p < 0.005$). No significant gender differences were noted regarding training at a medium level of effort (Fig. 1).

### Body Complaints

As shown in Table 1, 61% of the study group reported body complaints during the last 3 months, a higher number of females than males ($p = 0.03$). The most common body parts for complaints were the back, knees, and neck (Table 2). A higher number of females than males reported complaints from the back ($p = 0.002$), the knees ($p = 0.015$), the neck ($p = 0.001$), and the hip ($p = 0.015$) (Table 2).

#### *Self-Related Health and Body Complaints*

The reported self-related health did not correspond with body complaints in males. There were no differences between students in academic programs compared to those in vocational programs, or between students living in the countryside with those living in the city. A higher number of females with body complaints reported poorer health than those females without any complaints. A higher number of female students in academic programs and who were living in the city reported poorer health than those female students living in the countryside.

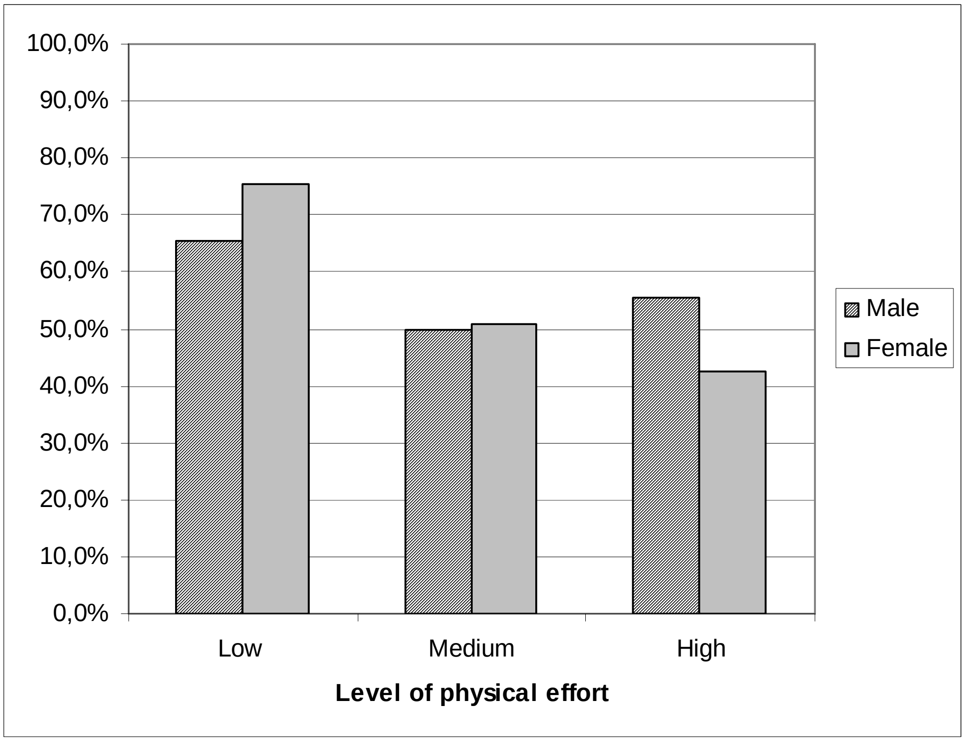

**FIGURE 1.** Level of physical effort reported by males and females (male, n = 484; female, n = 502, 7 people missing).

**TABLE 2**
**Students Who Reported Body Pain During the Last 3 Months**
**(n = 600, 8 People Missing)**

| Location | Male; n (%) | Female; n (%) | *p*-Value |
|---|---|---|---|
| Back | 152 (32) | 213 (42) | 0.002 |
| Knee | 115 (24) | 157 (31) | 0.015 |
| Neck | 67 (14) | 139 (27) | 0.001 |
| Ankle | 86 (18) | 78 (15) | ns |
| Shoulder | 52 (11) | 74 (15) | ns |
| Hand | 50 (10) | 44 (9) | ns |
| Hip | 22 (5) | 43 (8) | 0.015 |
| Elbow | 28 (6) | 19 (4) | ns |

## Neck Pain

Neck pain affected self-related health negatively ($p < 0.0005$). There was also a higher number of students in academic programs compared to vocational programs that reported neck pain ($p = 0.019$), and a higher number of females than males that reported neck pain ($p < 0.0005$). There were no differences between students living in the countryside compared to those living in the city in terms of neck pain. Table 3 shows that females suffering from neck pain estimated their self-related health as much worse than males with neck pain ($p = 0.017$).

**TABLE 3**
**Self-Related Health and Its Relation to Appearance of Neck Pain and Back Pain (n = 606, 2 People Missing)**

| | | Poor; n (%) | Neither Good nor Poor; n (%) | Good; n (%) | Total; n (*p*-Value) |
|---|---|---|---|---|---|
| No neck pain | Male | 8 (4) | 32 (15) | 169 (81) | 209 |
| | Female | 12 (6) | 37 (19) | 143 (75) | 192 (ns) |
| Neck pain | Male | 2 (3) | 15 (23) | 49 (74) | 66 |
| | Female | 13 (9) | 51 (37) | 75 (54) | 139 (0.017) |
| No back pain | Male | 2 (2) | 19 (14) | 105 (84) | 126 |
| | Female | 7 (6) | 20 (17) | 90 (77) | 117 (ns) |
| Back pain | Male | 8 (5) | 28 (19) | 114 (76) | 150 |
| | Female | 18 (8) | 68 (32) | 127 (60) | 213 (0.006) |

### *Back Pain*

Back pain affected self-related health negatively ($p = 0.018$). Of those reporting back pain, a higher number of females indicated poorer self-related health than males ($p = 0.006$) (Table 3). There were no differences between academic and vocational programs, or between students living in the countryside compared to those living in the city regarding back pain.

Among females reporting back pain, those in academic programs reported poorer self-related health than those in vocational programs.

### *Knee Pain*

Knee pain did not affect self-related health ($p = 0.515$). There were no significant differences between students living in the city compared to those living in the countryside, or between those in academic programs compared to students in vocational programs.

### Self-Related Health and Level of Physical Effort

Poor self-related health and a lower level of physical effort was related ($p < 0.001$) (Table 4). The correlation between self-related health and effort was 0.219 ($p < 0.01$). Table 4 shows that students reporting no existing, irregular, or low effort of physical activity (once or less per week) report their self-related health to be poor to a greater extent than those with a medium or high effort (two times or more per week).

A higher number of students who did not perform any physical activity reported neck pain ($p = 0.002$) (Table 5). Neck pain was reduced by increased physical activity. A lower number of students who performed physical activities at a high level compared to a medium or low level reported neck pain. No such differences were noted regarding back pain and knee pain.

## DISCUSSION

In the present study, 26% of the high school students participated in sports on a regular basis comprising no gender differences. In 16- to 18-year-old Finnish youths, Aarnio et al.[17] found that those who participated

**TABLE 4**
**Self-Related Health and Its Relation to Level of Exertion (n = 977, 16 People Missing)**

| Total Exertion | Poor; n (%) | Neither Good nor Poor; n (%) | Good; n (%) | Total, $x^2$; n (%) |
|---|---|---|---|---|
| Never/irregular/low | 25 (54) | 84 (45) | 208 (28) | 317 (32) |
| Medium/high | 21 (46) | 104 (55) | 535 (72) | 660 (68); $p$ = 0.001 |
| Total | 46 (100) | 188 (100) | 743 (100) | 977 (100) |

**TABLE 5**
**Level of Exertion by Students that Reported Neck Pain During the Last 3 Months (n = 600, 8 People Missing)**

| Total Exertion | Neck Pain | | Total, $x^2$ |
|---|---|---|---|
| | No | Yes | |
| Never/irregular | 19 | 23 | 42 |
| Low | 90 | 55 | 145; $p$ = 0.002 |
| Medium | 251 | 115 | 366 |
| High | 38 | 9 | 47 |
| Total | 398 (66%) | 202 (34%) | 600 (100%) |

in organized sports were more often persistently fit than those who did not. Adolescents should be encouraged to participate and try out different types of sports, and participate in organized sports. Leisure-time physical activity possibilities to participate in organized sports without competition should also be developed for those unwilling to compete in sports[17]. Aarnio et al. reported 24–28% of the girls and 32–36% of the boys to be extremely physically active[17]. This is somewhat more than in the present study. The 74% of the students who did not participate in sports reported performance of physical activity at a low or a medium level of physical effort. This might mean that they probably walked or went by bike to school, and when they spend time with friends.

General epidemiological findings concerning physical activity in young individuals suggest that boys were more active than girls, and that the amount of physical activity declined with increased age and also that this rate of decline was greater in girls than boys[18]. In the present study, we found that a significantly higher number of females compared to males participated in physical activities at a lower level of effort and a significantly higher number of males trained at a higher level of effort. In a longitudinal growth and health study from the Netherlands, the authors reported different levels of intensity when it comes to gender, where females were spending more time with activities at a medium level of effort than males. Both in males and females, there was a significant decrease in time spent on vigorous activities. In males, a decrease was found in time spent on extremely vigorous activities whereas in females, time spent at this level of activity remained more or less stable[18]. In another epidemiological study, it was reported that males are about 25% more fit than females[19]. Furthermore, during the school age, a consistent decline in physical activity has been noted, with males decreasing about 2.7% per year and females about 7.4% per year. These data suggest that older youth and females are at increased risk of obesity due to a sedentary lifestyle[19]. In our study, we could not find that males were more fit than females, but males participated in activities at a higher level of effort than females, which probably suggests that more males take part in competitive sports and females in recreation sports.

Males reported significantly better health than females; 81% of the males and 71% of the females reported their health to be good. Our results were better than those presented by Brattberg, who studied 471 Swedish school children in a younger age group (8–17 years old) and reported that 65% of those described themselves to be healthy. One reason might be that Brattberg studied health from a psychological as well as a physiological perspective, and made a combination of both[20]. The general consistency of results in different studies is impressive, although many studies differ with regard to their design, population, and the way in which self-related health is assessed[21]. The research into determinants for self-related health reveals that the global health status measures physical health problems and functional ability, as well as social and mental well-being[21]. Furthermore, the measure provides a succinct way of summarizing the diverse components under the broader health status rubric. Musculoskeletal symptoms are present early in school age students and it has been suggested that these early symptoms might be the start of a lifelong course of musculoskeletal complaints[21].

Of our study group, 61% reported that they have had body complaints during the last 3 months, representing a significantly higher number in females than males. The most common body parts for complaints were the back, knees, and neck. Very similar to our results were those of Smedbråten et al.[22], which showed that about 50% of the 15-year-old adolescents reported body complaints, and that knee and back pain were most frequently reported among adolescents. Females also reported more neck pain than males. These findings are also consistent with findings in the adult population[22].

Neck pain affected self-related health negatively. This result was similar to that reported by Cho et al.[23]. They found that the prevalence of musculoskeletal symptoms, especially from the neck region, was high in the adolescent population and demonstrated a certain association with psychological distress. High psychological distress, therefore, appears to be a contributing factor related to the musculoskeletal symptoms in adolescents[23].

There is a variety of reports about back pain in youth. Earlier epidemiological studies in general populations focusing on the age group of 16–20 years have shown a history of back pain in 20–66% of the individuals[20,22,24,25,26]. In the present study, the prevalence of back pain was 35% for males and 45% for females. Furthermore, back pain affected self-related health negatively. Of those reporting back pain, a higher number of individuals indicated poor self-related health. This result was similar to that of a Canadian study, where a positive association between poor mental health and development of low back pain was found[27]. Moreover, the results of the incidence of low back pain are comparable with those in the scientific literature.

It is often stated that postural defects in adults do not play any important role in terms of low back pain and in most patients the exact etiology of the pain remains unknown. One reason for developing chronic low back pain seems to be a combination of inappropriate treatment and sociopsychological factors. The possible etiological role of psychological factors has been discussed in both grown-up individuals and children[26].

In general, we could not find any differences between students in academic programs compared to those in vocational programs, or between students living in the countryside with those living in the city. We found only that a higher number of students in academic programs compared to vocational programs reported neck pain. Furthermore, a higher number of female students in academic programs who were living in the city reported poor health than females living in the countryside, and that neck and back pain affected self-related health negatively. This is in contrast to earlier studies[28,29] and the differences might depend on old studies, and that the climate at school has been more tough, demanding, and stressful in academic programs. Physical inactivity has emerged as an important risk factor for many chronic diseases and physical activity has been defined as “any bodily movement produced by skeletal muscles that result in energy expenditure”[30]. Physical activity might prevent body complaints as was predicted in a Norwegian study supporting less low back pain in children who walked to school compared with those who were brought to school by car or public transport[31].

## CONCLUSIONS

The results showed a high prevalence of musculoskeletal symptoms in this adolescent population and demonstrated a certain association with self-related health. Those students who did not participate in sports on a regular basis have even reported that they performed physical activity at a low or a medium level of physical effort. This might mean that they walked or went by bike to school, and when they spent time with friends. Therefore, it is important to make it easy for school children and adolescents to perform physical activity at school and during leisure time in order to prevent chronic diseases and, in addition, encourage them to take responsibility for their own well-being.

## ACKNOWLEDGMENTS

This research was supported by the Swedish National Center for Research in Sport (CIF), Stockholm, Sweden.

## References

1. Paffenbarger, R.S., Blair, S.N., and Lee, I.M. (2001) A history of physical activity, cardiovascular health and longevity: the scientific contributions of Jeremy N Morris. *Int. J. Epidemiol.* **30,** 1184–1192.
2. (1996) Physical Activity and Health: A Report of the Surgeon General: US Departments of Health Human Services. Centers for Disease Control and Prevention, Atlanta, GA.
3. Hardman, A.E. (2001) Physical activity and health: current issues and research needs. *Int. J. Epidemiol*. **30**, 1193–1197.
4. Anderson, J.J. (2000) The important role of physical activity in skeletal development: how exercise may counter low calcium intake. *Am. J. Clin. Nutr*. **71**, 1384–1386.
5. Duppe, H., Gardsell, P., Johnell, O., et al. (1997) Bone mineral density, muscle strength and physical activity: a population based study of 332 students aged 15–42 years. *Acta Orthop. Scand.* **68,** 97–103.
6. Barnekow-Bergkvist, M., Hedberg, G., Janlert, U., and Jansson, E. (1998) Prediction of physical fitness and physical activity level in adulthood by physical performance and physical activity in adolescence – an 18-year follow–up study. *Scand. J. Med. Sci. Sports* **8,** 299–308.
7. Engström, L.M. (1996) Worldwide trends in youth sport. In Engström, L.M. and Weiss, M., Eds. Human Kinetics, Champaign, IL. pp. 231–244.
8. Wendell, C.T., Blair, S.N., Cummings, S.S., et al. (1999) Childhood and adolescent physical activity patterns and adult physical activity. *Med. Sci. Sports Exerc*. **31,** 118–123.
9. Telama, R., Yang, X., Laakso, L., and Viikari, J. (1997) Physical activity in childhood and adolescence as predictor of physical activity in young adulthood. *Am. J. Prev. Med.* **13,** 317–332.
10. Westerstahl, M., Barnekow-Bergkvist, M., Hedberg, G., and Jansson, E. (2003) Secular trends in body dimensions and physical fitness among adolescents in Sweden from 1974 to 1995. *Scand. J. Med. Sci.* **13,** 128–137.
11. Raustorp, A., Ståhle, A., Gudasic, H., et al. (2005) Physical activity and self-perception in school children assessed with the children and youth-perception profile. *Scand. J. Med. Sci. Sports* **15**, 126–134.
12. Vincent, S.D., Pangrazi, R.P., Raustorp, A., et al. (2003) Activity levels and body mass index of children in the United states, Sweden and Australia. *Med. Sci. Sport Exerc*. 1367–1373.
13. Sallis, J.F. and Patrick, K. (1994) Physical activity guidelines for adolescents: consensus statement. *Pediatr. Exerc. Sci*. **6,** 302–314.
14. Klasson-Heggeb∅, L. and Anderssen, S.A. (2003) Gender and age differences in relation to the recommendations of physical activity among Norwegian children and youth. *Scand. J. Med. Sci. Sports* **13**, 293–298.
15. Brattberg, G. and Wickman, V. (1992) Prevalence of back pain and headache in Swedish school children: a questionnaire survey. *Pain Clin*. **5(4),** 211–220.
16. Alricsson, M. and Werner, S. (2005) Self-reported health, physical activity and prevalence of complaints in elite cross-country skiers and matched controls. *J. Sports Med. Phys. Fitness* **45(4),** 547–552.
17. Aarnio, M., Winter, T., Peltonen, J., Kujala, U.M., and Kaprio, J. (2002) Stability of leisure-time physical activity during adolescence - a longitudinal study among 16-, 17- and 18-years-old Finnish youth. *Scand. J. Med. Sci. Sports* **12,** 179–185.
18. Mechelen, W.V., Twisk, J.W.R., Post, G.B., et al. (2000) Physical activity of young people: the Amsterdam longitudinal growth and health study. *Med. Sci. Sports Exerc*. **32(9),** 1610–1616.
19. Sallis, J.F. (1993) Epidemiology of physical activity and fitness in children and adolescents. *Food Sci. Nutr*. **33**, 403–

408.
20. Brattberg, G. (1994) The incidence of back pain and headache among Swedish school children. *Qual. Life Res*. **3**, 27–31.
21. Undén, A.L. and Elofsson, S. (2001) Health from the patient's point of view. How does it relate to the physician's judgement? *Fam. Pract*. **18,** 174–180.
22. Smedbråten, B.K., Natvig, B., and Rutle, O. (1998) Self-reported bodily pain in schoolchildren. *Scand. J. Rheumatol*. **27**, 273–276.
23. Cho, C.-Y., Hwang, I.-S., and Chen, C.C. (2003) The association between psychological distress and musculoskeletal symptoms experienced by Chinese high school students. *J. Orthop. Sports Phys. Ther*. **33**, 344–353.
24. Kujala, U.M., Taimela, S., and Viljanen, T. (1999) Leisure physical activity and various pain symptoms among adolescents. *Br. J. Sports Med*. **33**, 325–328.
25. Balagué, F., Nordin, M., and Skovron, M.L. (1994) Non-specific low-back pain among schoolchildren: a field survey with analysis of some associated factors. *J Spinal Disord*. **7(5),** 374–379.
26. Balagué, F., Dutoit, G., and Waldburger, M. (1988) Low back pain in schoolchildren. *Scand. J. Rehab. Med*. **20**, 175–179.
27. Feldman, D.E., Shrier, I., Rossignol, M., and Abenhaim, L. (2001) Risk factors for the development of low back pain in adolescence. *Am. J. Epidemiol*. **154**, 30–36.
28. Andersen, L.B. and Schelin, B. (1994) Physical activity and performance in a random sample of adolescents attending school in Denmark. *Scand. J. Med. Sci. Sports* **4,** 13–18.
29. Aarnio, M., Kujala, U.M., and Kaprio, J. (1997) Associations of health-related behaviours, school type and health status to physical activity patterns in 16 year old boys and girls. *Scand. J. Soc. Med*. **25**, 156–167.
30. Caspersen, C.J., Powell, K.E., and Christenson, G.M. (1985) Physical activity, exercise, and physical fitness: definitions and distinctions for health-related research. *Public Health Rep.* **100**, 126–131.
31. Sjolie, A.N. (2004) Associations between activities and low back pain in adolescents. *Scand. J. Med. Sci. Sports* **14**, 352–359.

## BIOSKETCHES

**Marie Alricsson, PhD, RPT,** is a physiotherapist who graduated from the Karolinska Institutet, Stockholm with her PhD in sports medicine. Her research and publications are in the area of public health, prevention, sports medicine, and rehabilitation. She is working as a researcher and a senior lecturer in rehabilitation science at the Mid Sweden University, Department of Health Sciences, SE- 831 25 Östersund, Sweden. E-mail: marie.alricsson@miun.se

**Bodil J. Landstad**, **PhD,** is a social scientist who graduated from the Mid Sweden University in Östersund. She has a PhD in rehabilitation medicine from the Karolinska Institute in Stockholm, Department of Rehabilitation Medicine. Her research is mainly in the area of public health, prevention, and early rehabilitation at the workplace. Dr. Landstad has broad experience from the rehabilitation area and she has, together with colleagues, initiated and developed a bachelor degree, a master degree, as well as PhD studies in rehabilitation science at the Mid Sweden University. She is working part time as a senior lecturer in rehabilitation science at the Mid Sweden University, Department of Health Sciences, and part time as a senior lecturer in health science at Nord-Trøndelag University College, Norway. E-mail: bodil.landstad@miun.se

**Ulla Romild**, **PhD,** is a statistican and a graduate of Uppsala University. Her research has mainly been in the field of Tourism and Leisure Studies. She is a lecturer at the Department of Information Technology and Media at Mid Sweden University. E-mail: ulla.romild@miun.se

**Suzanne Werner**, **PhD, RPT, ATC** is an associate professor and university lecturer at the Karolinska Institutet, Stockholm, Sweden. She is the vice president of the International Study Group of Proprioception and a member of the advisory boards of the Swedish Research Council in Sports Medicine; the European Rehabilitation Panel; the Sports Medicine and Sports Surgery Center, Mount Alvernia Hospital, Singapore; and a member of the editorial boards of *Physical Therapy in Sports* and *Journal of Orthopaedic and Sports Physical Therapy*. Her research and publications are in the area of orthopedics, sports medicine, and sports medicine rehabilitation. She has the position as the chief coordinator responsible for research and education in sports medicine and sports rehabilitation at Capio Artro Clinic, Stockholm Sports Trauma Research Center, Karolinska Institutet. E-mail: suzanne.werner@capio.se
